# Atypical Morphological Variations of the Sacrum in the Korean Population: A PMCT-Based 3D Reconstruction Study

**DOI:** 10.3390/medicina61111942

**Published:** 2025-10-29

**Authors:** Jeong-Hyun Park, Eun-Seo Park, Jaeho Cho, Yu-Jin Choi, Hyung-Wook Kwon, Digud Kim, Yunil Choe, Goeun Lee, Kwang-Rak Park

**Affiliations:** 1 Department of Anatomy & Cell Biology, College of Medicine, Kangwon National University, Chuncheon-si 24341, Republic of Korea; jhpark@kangwon.ac.kr (J.-H.P.); police5565@hanmail.net (Y.-J.C.); kwenhw@naver.com (H.-W.K.); oe5235@naver.com (D.K.); loloo4ve@naver.com (Y.C.); 2 Department of Anatomy, College of Korean Medicine, Sangji University, Wonju-si 26339, Republic of Korea; pes1218@naver.com; 3 Department of Orthopaedic Surgery, Chuncheon Sacred Heart Hospital, Hallym University, Chuncheon-si 24253, Republic of Korea; hohotoy@nate.com; 4 Division of Forensic Medical Examination, National Forensic Service, 10, Ipchun-ro, Wonju-si 26460, Republic of Korea; delphini@korea.kr

**Keywords:** sacral variation, sacrum morphology, PMCT, sacralization

## Abstract

*Background and Objectives*: The sacrum is formed by five fused vertebrae and connects the lumbar spine to the coccyx. It has four pairs of foramina for sacral nerves and shows important anatomical variations. This study aims to analyze the frequency of atypical sacral morphology in the Korean population using 3D reconstruction of postmortem computed tomography (PMCT) images, and to provide a systematic classification and morphological characterization. *Materials and Methods*: A total of 29 PMCT datasets (10 males, 19 females) from the National Forensic Service were used to generate 3D sacral models with Mimics software for the analysis of atypical sacral morphology. Key morphometric parameters, including sacral width (SW), sacral length (SL), sacral foramina distances (SFD1, SFD2), sacral vertebral heights (SH1, SH2), sacral curvature (SC), and sacral index (SI), were measured. Sacral foramina were categorized into three groups based on completeness, and auricular surfaces were classified into three types according to their vertical position. *Results*: Median values for sacral dimensions were as follows: SW 95.3 mm, SL 118.6 mm, SFD1 36.1 mm, SFD2 28.8 mm, SH1 28.0 mm, SH2 29.7 mm, SC 0.92, and SI 0.78. Sacral foramina variations were identified in 12 of 29 cases (41.4%) as incomplete, including one case with an incomplete upper opening. No significant sex-based differences were found in foramen or auricular surface types, although females showed higher values for SW and SI (SW: 97.2 mm, SI: 0.86). Correlation analysis revealed positive associations between SL and both stature (r = 0.635) and weight (r = 0.645), and negative correlations between SI and stature (r = −0.663), SL (r = −0.921), and SC (r = −0.845). Two cases (6.8%) exhibited sacralization, while the remaining 25 cases had the configuration of five lumbar vertebrae and six sacral segments. *Conclusions*: Our findings support the notion that atypical segmentation patterns are more prevalent than sacralization. Atypical sacral morphology was observed in 29 cases (19.8%), most commonly involving a normal lumbar spine with six sacral segments. These findings highlight the relevance of sacral variation in clinical and anatomical contexts.

## 1. Introduction

The sacrum consists of five fused sacral vertebrae that are large and triangular in shape, forming the posterior wall of the pelvic cavity [1,2]. Its broad upper base articulates with the fifth lumbar vertebra at the lumbosacral angle, and its blunt caudal tip articulates with the coccyx. The normal sacrum contains four pairs of anterior and posterior sacral foramina, which communicate with the sacral canal and transmit the corresponding sacral nerves (S1–S4) [3].

One common anatomical variation related to the sacrum is the lumbosacral transitional vertebra (LSTV). This occurs either due to sacralization, where one or both transverse processes of the fifth lumbar vertebra fuse with the first sacral segment, or due to lumbarization, in which the first sacral segment displays an abnormal transverse process resembling that of a lumbar vertebra. Moreover, previous studies have shown that atypical segmentation occurs more frequently in the sacralization of the first coccygeal vertebra than in the sacral region [4,5].

Sacralization can cause various clinical problems, and LSTV is a common spinal variation observed in approximately 10–25% of the general population [6]. If the fifth lumbar vertebra fuses with the sacrum, there is a possibility of compression of the fifth sacral nerve (S5), which may result in sciatica and back pain. Recently, spinopelvic fixation surgery has rapidly increased, leading to growing research and interest in this area. In particular, unilateral lumbarization or sacralization can cause asymmetry in the dorsal sacral landmarks and may occasionally lead to hemipelvis elevation [7]. However, due to the lack of anatomical studies on sacralization, further research based on precise anatomical understanding is required. In particular, Viktoria et al. [4] recommended classifying the entire spine, including the sacrum.

Studies on sacral morphological variations have also been conducted in other countries. The prevalence was 58.1% in China [8], 21.1% in Bangladesh [9], 1.58% in Western India [2], and 12% in India [10]. There have been studies on the morphology of the sacrum and sacrococcygeal region in Korean populations [5,11]. However, studies that have comprehensively analyzed sacrum variations are very rare.

Modern medicine relies heavily on radiological examinations such as computed tomography (CT) or magnetic resonance imaging (MRI). Utilizing three-dimensional (3D) programs to analyze data from these modalities provides a more objective method. CT offers a comprehensive anatomical view and enables multiplanar and 3D reconstructions, which are especially beneficial for assessing the sacrum, which lies in a semicoronal plane. The high soft tissue-to-bone contrast in CT images facilitates clear visualization of the entire cortex, aiding in the detailed evaluation of fractures, structural damage to the sacroiliac joint in spondylarthritis, as well as the identification of osseous tumors [12,13,14]. According to Vrtovec et al. [15], the geometric relationships between the anatomical structures of the sacrum, pelvis, and hip joints—which define the sagittal alignment parameters of the pelvis—can be better observed in 3D images than in 2D images.

In clinical practice, the shape of the sacrum is closely related to several procedures, such as sacroiliac screw insertion, pedicle screw fixation, and epidural anesthesia. A clear anatomical understanding of its form and structure is therefore important for safe and effective surgical access. Yet, detailed morphometric analyses of atypical segmentation or unusual sacral patterns have rarely been reported [16].

Moreover, recent studies have suggested the potential use of regenerative biomaterials in spinal and pelvic reconstruction, particularly in cases involving structural variation or surgical resection. These biocompatible materials may contribute to soft tissue repair and guided tissue regeneration. In this context, integrating anatomical understanding of sacral morphology with regenerative approaches may provide a valuable foundation for future reconstructive applications [17].

This study aims to analyze the various morphological variations in the sacrum through 3D reconstruction of postmortem computed tomography (PMCT) images and to systematically classify and present these variations. Additionally, it seeks to establish a database of sacral variations in the Korean population to enhance clinical and anatomical understanding of sacral morphology and its deviations.

## 2. Materials and Methods

This study examined the sacra of 29 individuals (10 male, 19 female) from the Korean population, obtained at the National Forensic Service in 2022. Of the 152 individuals, 29 were selected based on the presence of sacralization or a variant form of the sacrum. A variant form was defined as having five or three sacral foramina. PMCT is widely utilized in forensic medicine for its non-invasive capability to visualize injuries, detect hemorrhages, and estimate volumes. The research was approved by the Ethics Committee of the National Forensic Service (Institutional Review Board number: 906-250319-HR-004-05). The average age of the subjects at the time of imaging was 54.0 years, with an age range of 30 to 78 years. PMCT images depicting fractures, injuries, deformities, or signs of prior surgery in the gluteal region were excluded. The study only included images from deceased individuals whose primary cause of death was not related to the gluteal region.

### 2.1. PMCT Image and Data Acquisition

**Figure 1:**
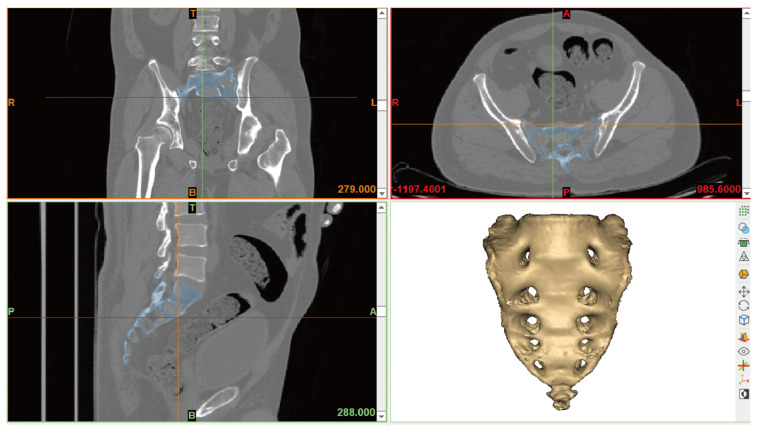
Three-dimensional reconstruction of postmortem computed tomography (PMCT) images using Mimics software (version 22.0).

In this study, examinations were performed using the PMCT (Aquilion PRIME TSX-303A, CANON Medical Systems Corp., Tokyo, Japan) equipment at the National Forensic Service headquarters. The imaging parameters were configured with a tube voltage of 120 kVp, a pitch factor of 0.637, a slice thickness of 1.0 mm, an increment of 0.8 mm, and a rotation time of 0.6 s. To enhance bone contrast and sharpness, all images were obtained using bone settings (window width: 1500 HU, window level: 500HU). The PMCT images were provided in the DICOM file format. For 3D modeling of the sacrum, Mimics (version 22.0, Materialise, Leuven, Belgium) software was used to create 3D models from the DICOM data based on coronal, sagittal, and axial planes. For the sacrum’s 3D image, Hounsfield Unit (HU) values, which correspond to grayscale, were modified in the 3D model. A new mask was created, and the threshold value was adjusted from a minimum of 180 HU to a maximum of 3071 HU to isolate the sacrum. To measure the sacrum, the generated mask was employed to create a sacrum object using the calculate part function (Figure 1).

### 2.2. Measurement of Sacrum Morphology

**Figure 2:**
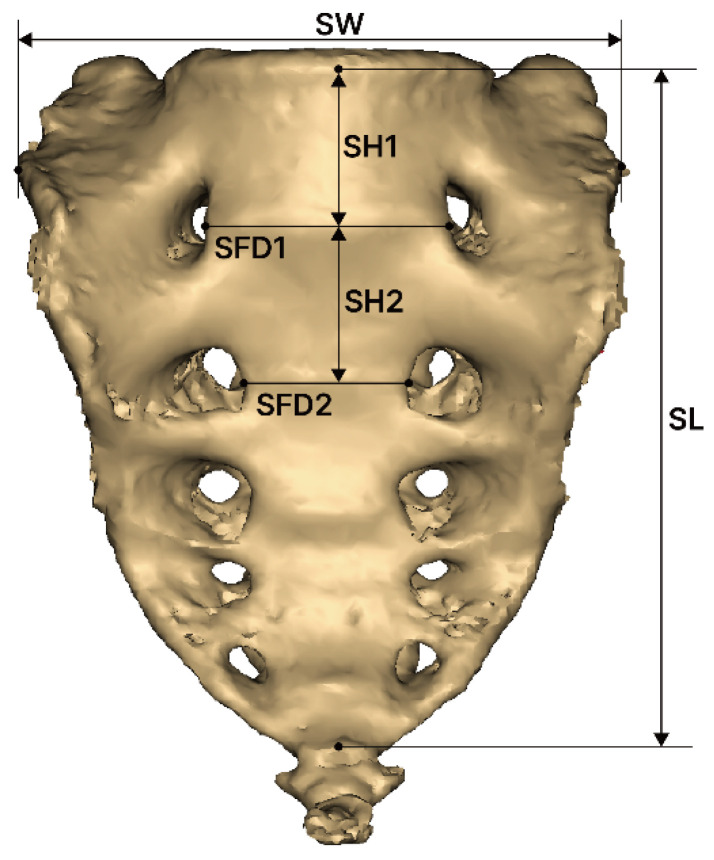
Morphometric measurement of the sacrum. SW, sacral width; SL, sacral length; SFD1, distance between the first pair of sacral foramina; SFD2, distance between the second pair of sacral foramina; SH1, height of the first sacral vertebra; and SH2, height of the second sacral vertebra.

Sacrum morphology measurements were conducted using Mimics software. To ensure accuracy and repeatability of the results and measurement variables and to minimize measurement errors, two researchers conducted the measurements independently. The definitions of the sacrum measurement variables are as follows, based on previous research [18] (Figure 2):

- SW (sacral width): Measured between the auricular surfaces at the posterosuperior point of the auricular surface.
- SL (sacral length): Measured from the upper midpoint of the promontory to the apex of the sacrum in the sagittal plane.
- SFD1 (sacral foramina distance of first): Measured between the left and right first anterior sacral foramina.
- SFD2 (sacral foramina distance of second): Measured between the left and right second anterior sacral foramina.
- SH1 (sacrum vertebrae height of first): Measured the height of the first sacral vertebra at the midline.
- SH2 (sacrum vertebrae height of second): Measured the height of the second sacral vertebra at the midline.
- SC (sacral curvature): Calculated as the quotient of the sacral anterior surface distance divided by SL.
- SI (sacrum index): Calculated as the quotient of SL divided by SW.

### 2.3. Grouping of Sacral Foraminal Variations

**Figure 3:**
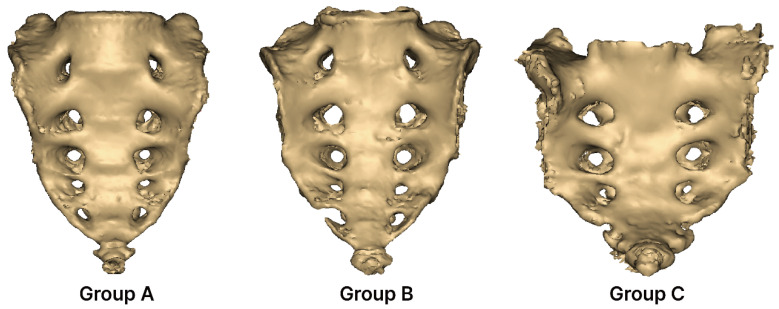
Classification of sacral foramina variations. Group A: none of the foramina are perforated or separated; all sides are completely closed. Group B: the foramina are not fully closed on either the right or left side and remain open. Group C: only three pairs of foramina are completely closed.

The variations in the sacral foramina were classified into three groups. The definition of each group is as follows (Figure 3):

- Group A (complete five pairs): Consists of five complete pairs of foramina. In Group A, none of the foramina are perforated or separated; all sides are completely closed.
- Group B (incomplete five pairs): Contains five incomplete pairs of foramina. In Group B, the foramina are not fully closed on either the right or left side and remain open.
- Group C (complete three pairs): Consists of three complete pairs of foramina. In Group C, only three pairs of foramina are completely closed.

### 2.4. Type of Sacral Auricular Surface

**Figure 4:**
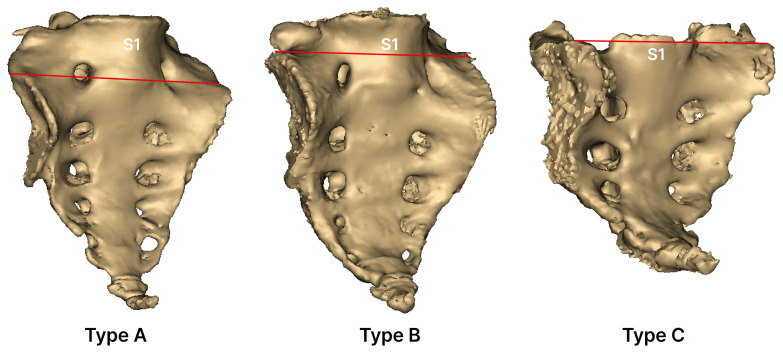
Types of sacral auricular surface based on vertical position. Type A: auricular surface extending from the lower part of S1; Type B: auricular surface extending from the upper part of S1; and Type C: auricular surface extending from a level above the upper part of S1.

This classification is based on the vertical location of the sacral auricular surface, following previous studies [19] (Figure 4):

- Type A (low-down): Auricular surface extending from the lower part of S1.
- Type B (standard): Auricular surface extending from the upper part of S1.
- Type C (high-up): Auricular surface extending from a level above the upper part of S1.

### 2.5. Statistical Analysis

All data processing and statistical analyses were conducted using IBM SPSS version 23.0 (IBM Co., Armonk, NY, USA). The intraclass correlation coefficient (ICC) was employed to assess the interclass reliability of the measurements. Based on the criteria established by Koo and Li [20], the ICC values were categorized as follows: “excellent” for values of 0.81 or higher, “good” for values between 0.61 and 0.80, “moderate” for values ranging from 0.41 to 0.60, and “poor” for values below 0.40. Descriptive analyses were performed to characterize the sacral measurement variables. Frequency analyses of the variation in sacral foramina groups and sacrum auricular surface types, categorized by sex, were conducted using Fisher’s exact test. Independent samples t-tests were used to compare the means of each measurement variable. Correlation analysis was performed to examine the relationship between all measured variables and stature, with higher Pearson correlation coefficients (R) indicating stronger associations. A *p*-value of <0.05 was considered statistically significant.

## 3. Result

The results of the interclass correlation coefficients for all measurements (SW, SL, SFD1, SFD2, SH1, SH2, SC) were as follows: ICC was 0.9 or excellent for all measured variables (Table 1). The results of the characteristics of sacral measurement variables (interquartile range, median) were as follows: stature was found to have a median value of 166.0 and an IQR of 153.5, 173.0; weight was found to have a median value of 62.0 and an IQR of 53.5, 79.5; SW was found to have a median value of 95.3 and an IQR of 91.6, 100.2; SL was found to have a median value of 118.6 and an IQR of 105.1, 129.8; SFD1 was found to have a median value of 36.1 and an IQR of 31.1, 40.5; SFD2 was found to have a median value of 28.8 and an IQR of 27.2, 30.9; SH1 was found to have a median value of 28.0 and an IQR of 23.3, 31.3; SH2 was found to have a median value of 29.7 and an IQR of 25.6, 34.3; SC was found to have a median value of 0.92 and an IQR of 0.90, 0.95; and SI was found to have a median value of 0.78 and an IQR of 0.74, 0.91(Table 2).

The results of the classification of sacral foramina groups by sex were as follows: there was no significant difference in sacral foramina by sex (*p* = 0.629); group A consisted of 7 males and 9 females, totaling 16 (55.2%); group B consisted of 3 males and 9 females, totaling 12 (41.4%); and group C consisted of 0 males and 1 female, totaling 1 (3.4%) (Table 3). The results of the classification of sacral auricular surfaces by sex were as follows: there was no significant difference in sacral auricular surface by sex (*p* = 0.805); type A included 7 males and 11 females, totaling 18 cases (62.1%); type B included 3 males and 7 females, totaling 10 cases (34.5%); and type C included 0 males and 1 female, totaling 1 case (3.4%) (Table 4).

The results of the comparison of mean sacral measurement parameters by sex were as follows: SW in females (97.2 ± 5.4) had higher values than in males (93.0 ± 5.0), with a significant result observed (*p* = 0.49); SI in females (0.86 ± 0.15) had higher values than in males (0.75 ± 0.05), with a significant result observed (*p* = 0.007) (Table 5). The results of the correlation analysis of sacral measurement variables were as follows: there was a correlation in most of the variables. There was a positive correlation between SL and stature (r = 0.635) and weight (r = 0.645), all with *p* < 0.001. There was a positive correlation between SH2 and SL (r = 0.605) and SFD1(r = 0.683), all with *p* < 0.001. There was a negative correlation between SI and stature (r = −0.663), weight (r = −0.661), SL (r = −0.921), and SC (r = −0.845), all with *p* < 0.001 (Table 6).

## 4. Discussion

In this study, morphological variation of the sacrum was identified in 29 out of 152 specimens, corresponding to a frequency of approximately 19.8%. This finding showed a similar range to previous anatomical studies, such as a study conducted in India [21], which reported a prevalence of 28.6% (63 out of 220 cases), and another study from Bangladesh [9], which reported 21.1% (46 out of 218 cases). Although these studies yielded results comparable to ours, existing data are still limited in terms of ethnic and geographical diversity. Therefore, further comparative research across different populations is warranted to establish whether such variations represent a consistent anatomical feature or exhibit population-specific patterns. Recognizing the frequency and patterns of sacral morphological variation holds clinical relevance, as it may influence anatomical interpretation in medical imaging and decision-making during procedures involving the lumbosacral region. Accurate identification of atypical segmentation is particularly important given its potential association with disk herniation, degenerative joint disorders, and low back pain. Enhanced morphometric understanding may ultimately contribute to safer and more individualized clinical approaches.

In our study, we divided the groups according to sacral foramina and analyzed them. Of the 29 cases, 12 were incompletely opened. Of them, only one was open at the upper part of the sacrum. In this one case, complete fusion was observed at the lower part of the sacrum, which was a small number at 3.4% of the total. Similarly, in a previous study [21], 8 out of 60 cases (13.3%) were opened at the upper portion on one side. In this way, it seemed rare for L5 and S1 to fuse and have an incomplete opening on one side. In addition, since the variant form has a significant impact on the clinical situation in which the nerve exits through the sacral foramen, further analysis of this variant form is necessary.

In our study, 12 out of 29 variations in the sacral foramen were incomplete. Among them, 1 was incompletely opened in the upper portion and 11 (37.9%) were incompletely opened in the lower portion. A Bangladeshi study [9] reported that the frequency of sacralization opening from the upper (lumbar) or lower (coccyx) portion was 15 (32.6%) on the lumbar side and 31 (67.3%) on the coccyx side. In our study and a previous study, there were more cases of incomplete opening from the lower than from the upper portion.

According to a previous study [4], when an additional vertebral segment is present, it may shift or transform into another region, resulting in partial or complete fusion of the last lumbar vertebra with the sacrum at the lumbosacral junction; such cases are classified as atypical sacral morphologies. In addition, various atypical segmental morphologies were observed in the coccyx in that study, highlighting the need for further investigation. However, research on coccygeal sacralization has not been sufficiently conducted yet, so further research is needed [22].

According to Shaheen et al. [23], coccygeal sacralization refers to the fusion of the first coccygeal vertebra (Co1) with the fifth sacral vertebra (S5), which may give the appearance of a sacrum comprising six vertebrae. In addition, Lee et al. [22] reported that synostosis between Co1 and S5 occurs frequently and is often associated with variations in the number of sacral vertebrae. These findings suggest that the presence of six sacral segments does not always indicate sacralization in the strict sense but may result from coccygeal fusion, emphasizing the need for careful differentiation. In our study, among the 29 variations, 2 cases (6.8%) were identified as sacralization, characterized by the presence of four lumbar and six sacral segments. The remaining 25 cases (86.2%) had five lumbar and six sacral segments and were considered atypical segmentations. In a previous study [4], 5 cases (8.3%) were classified as sacralization, while 60 out of 145 cases (41.3%) were identified as atypical segmentations. These results support the conclusion that sacralization is relatively uncommon, whereas atypical segmental patterns are more frequent. However, many studies [4,9,24] have defined sacralization based only on increased sacral segment numbers, without considering the lumbar count, potentially leading to inaccurate prevalence data. A more precise definition should account for both lumbar and sacral vertebral numbers to avoid such misclassification.

In this study, the morphological analysis results of the sacral auricular surface type showed that low-down was the most common with 18 types (62.1%), followed by standard with 10 types (34.5%), and high-up with 1 type (3.4%). The high-up type had a three-pair sacrum, which was the smallest variant form among all, and the auricular surface was also a rare form. In our study, the form in which the sacral foramina were fused at the top was mainly observed. In a previous study [25], the type classification analysis results showed that standard (217 cases), low-down (44 cases), and high-up (39 cases) were the most common, in that order. While our study had many low-down forms, the previous study had more standard forms. Since the morphological analysis was conducted on the variant forms in this study, it seems to have affected the sacral auricular surface.

In our study, measurement was performed where the mean SH1 value was 27.8 ± 4.5, and the mean SH2 value was 29.5 ± 5.1. The mean SH1 value in the South African population [18] was 31.83 ± 3.78, and the mean SH2 value was 26.39 ± 3.65. The SH1 value in the Indian population [26] was 29.62 ± 2.65, and the SH2 value was 25.45 ± 2.41. When comparing our study with previous studies, the measured SH1 value of our study was smaller than previous studies, and the SH2 value was larger than previous studies. Since the previous studies measured the normal sacrum, the observed differences showed different results by race, and it is thought that further research is necessary.

The present study has several limitations. First, because 3D reconstruction techniques were used, discrepancies may exist compared to direct measurements on actual human sacra. If necessary, future studies involving cadaveric specimens should be conducted to validate these findings. Second, this study focused on morphological variations and included a relatively small sample size of 29 subjects. These cases were selected based on the presence of identifiable atypical morphologies, rather than for prevalence analysis. The observed frequency of such variations was 19.8%. Further studies involving larger populations are needed to improve the statistical robustness and generalizability of these findings.

## 5. Conclusions

This study analyzed morphological variations in the sacrum and found that 29 cases (19.8%) exhibited atypical morphologies. The most frequent pattern involved a normal lumbar spine with six sacral segments, observed in 25 cases. These results underscore the importance of recognizing sacral variation in anatomical and clinical contexts.

## Figures and Tables

**Table 1:** Interclass correlation coefficients for all measurements.

Parameter	Interclass Correlation	
	ICC	95% CI
SW	0.980	0.98~0.991
SL	0.998	0.996~0.999
SFD1	0.997	0.994~0.999
SFD2	0.977	0.950~0.989
SH1	0.979	0.955~0.990
SH2	0.991	0.982~0.996
SC	0.977	0.950~0.989
SI	0.996	0.992~0.998

CI, confidence interval; SW, sacral width; SL, sacral length; SFD1, sacral foramina distance of first; SFD2, sacral foramina distance of second; SH1, sacrum vertebrae height of first; SH2, sacrum vertebrae height of second; SC, sacral curvature; and SI, sacral index.

**Table 2:** Characteristics of sacral measurement variables (interquartile range, median, and range).

	Male (*n* = 10)		Female (*n* = 19)		Total (*n* = 29)	
Parameter	Median (IQR)	Range	Median (IQR)	Range	Median (IQR)	Range
Age	51.5 (46.7, 46.5)	30 to 61	59.0 (44.0, 68.0)	41 to 81	54.0 (46.5, 64.0)	30.0 to 78.0
Stature (cm)	173.0 (172.2, 176.2)	168.0 to 1800.0	155.0 (150.0, 166.0)	146.0 to 168.0	166.0 (153.5, 173.0)	146.0 to 180.0
Weight (kg)	76.5 (67.7, 83.2)	56.0 to 90.2	54.0 (51.0, 65.0)	37.0 to 89.0	62.0 (53.5, 79.5)	37.0 to 90.2
SW (mm)	93.2 (87.6, 97.4)	85.9 to 101.4	96.8 (92.2, 102.9)	90.2 to 105.8	95.3 (91.6, 100.2)	85.9 to 105.8
SL (mm)	126.6 (115.9, 129.1)	114.7 to 131.2	118.6 (95.8, 131.1)	86.1 to 142.19	118.6 (105.1, 129.8)	86.1 to 142.1
SFD1 (mm)	37.7 (34.2, 41.2)	29.6 to 46.1	35.9 (30.4, 40.7)	25.9 to 45.4	36.1 (31.1, 40.5)	25.9 to 46.1
SFD2 (mm)	30.3 (28.2, 31.2)	27.5 to 31.8	28.7 (27.6, 30.2)	23.9 to 35.6	28.8 (27.2, 30.9)	23.9 to 35.6
SH1 (mm)	28.5 (24.1, 32.6)	22.1 to 35.3	28.0 (23.2, 30.6)	19.2 to 34.8	28.0 (23.3, 31.3)	19.2 to 35.3
SH2 (mm)	28.5 (25.2, 33.8)	22.9 to 34.7	30.7 (25.7, 35.1)	20.2 to 36.2	29.7 (25.6, 34.3)	20.2 to 36.2
SC	0.93 (0.91, 0.95)	0.91 to 0.96	0.91 (0.89, 0.95)	80.0 to 98.0	0.92 (0.90, 0.95)	0.80 to 0.98
SI	0.75 (0.71, 0.77)	0.65 to 0.85	0.79 (0.76, 1.01)	0.65 to 1.13	0.78 (0.74, 0.91)	0.65 to 1.13

IQR, interquartile range; SW, sacral width; SL, sacral length; SFD1, sacral foramina distance of first; SFD2, sacral foramina distance of second; SH1, sacrum vertebrae height of first; SH2, sacrum vertebrae height of second; SC, sacral curvature; and SI, sacral index.

**Table 3:** Classification of sacral foramina groups by sex.

Group	Male	Female	Total	*p*
Group A (Complete five pairs)	7 (70.0)	9 (47.4)	16 (55.2)	0.629
Group B (Unilateral five pairs)	3 (30.0)	9 (47.4)	12 (41.4)	
Group C (Complete three pairs)	0 (0.0)	1 (5.3)	1 (3.4)	
Total	10 (100.0)	19 (100.0)	29 (100.0)	

The data are presented as number (percent).

**Table 4:** Classification of sacral auricular surface types by sex.

Type	Male	Female	Total	*p*
Type A (Low-down)	7 (70.0)	11 (57.9)	18 (62.1)	0.805
Type B (Normal)	3 (30.0)	7 (36.8)	10 (34.5)	
Type C (High-up)	0 (0.0)	1 (5.3)	1 (3.4)	
Total	10 (100.0)	19 (100.0)	29 (100.0)	

The data are presented as number (percent).

**Table 5:** Comparison of mean sacral measurement parameters by sex.

Parameter (mm)	Male (*n* = 10)	Female (*n* = 19)	Total (*n* = 29)	*p*
SW	93.0 ± 5.0	97.2 ± 5.4	95.8 ± 5.5	0.049
SL	124.0 ± 6.2	115.1 ± 17.9	118.2 ± 15.4	0.064
SFD1	37.6 ± 5.0	35.1 ± 5.9	35.9 ± 5.6	0.271
SFD2	29.8 ± 1.5	28.9 ± 2.7	29.2 ± 2.4	0.352
SH1	28.5 ± 4.6	27.3 ± 4.5	27.8 ± 4.5	0.506
SH2	29.1 ± 4.3	29.7 ± 5.5	29.5 ± 5.1	0.753
SC	0.93 ± 0.01	0.90 ± 0.05	0.91 ± 0.04	0.070
SI	0.75 ± 0.05	0.86 ± 0.15	0.82 ± 0.13	0.007

The data are expressed by mean ± standard deviation. SW, sacral width; SL, sacral length; SFD1, sacral foramina distance of first; SFD2, sacral foramina distance of second; SH1, sacrum vertebrae height of first; SH2, sacrum vertebrae height of second; SC, sacral curvature; and SI, sacral index.

**Table 6:** Correlation analysis of sacral measurement variables.

	Stature	Weight	SW	SL	SFD1	SFD2	SH1	SH2	SC
Weight	0.710 **								
SW	−0.273	−0.237							
SL	0.635 **	0.645 **	−0.129						
SFD1	0.157	0.276	0.032	0.332					
SFD2	0.103	0.164	0.082	0.193	0.704 **				
SH1	0.242	−0.156	0.149	0.003	−0.654 **	−0.441 *			
SH2	0.184	0.338	−0.02	0.605 **	0.683 **	0.377 *	−0.505 **		
SC	0.543 **	0.666 **	−0.463 *	0.764 **	0.181	0.145	−0.063	0.406 *	
SI	−0.663 **	−0.661 **	0.493 **	−0.921 **	−0.303	−0.14	0.065	−0.547 **	−0.845 **

* *p* < 0.05 and ** *p* < 0.001. SW, sacral width; SL, sacral length; SFD1, sacral foramina distance of first; SFD2, sacral foramina distance of second; SH1, sacrum vertebrae height of first; SH2, sacrum vertebrae height of second; SC, sacral curvature; and SI, sacral index.

## Data Availability

The data used to support the findings of this study are available from the corresponding author upon request.

## Abbreviations

PMCT	Postmortem computed tomography
LSTV	Lumbosacral transitional vertebra
CT	Computed tomography
SW	Sacral width
SL	Sacral length
SFD1	Distance between the first pair of sacral foramina
SFD2	Distance between the second pair of sacral foramina
SH1	Height of the first sacral vertebra
SH2	Height of the second sacral vertebra
SC	Sacral curvature
SI	Sacral index
